# Dogs are the main species involved in animal-vehicle collisions in southern Spain: Daily, seasonal and spatial analyses of collisions

**DOI:** 10.1371/journal.pone.0203693

**Published:** 2018-09-14

**Authors:** David Canal, Beatriz Martín, Manuela de Lucas, Miguel Ferrer

**Affiliations:** 1 Applied Ecology Group, Estación Biológica de Doñana (EBD-CSIC), c/Américo Vespucio s/n, Seville, Spain; 2 Centro para el Estudio y Conservación de las Aves Rapaces en Argentina (CECARA-UNLPam) & Instituto de las Ciencias de la Tierra y Ambientales de La Pampa (INCITAP), Consejo Nacional de Investigaciones Científicas y Técnicas (CONICET), Santa Rosa, Argentina; 3 Fundación Migres, Centro Internacional de Migración de Aves -International Bird Migration Center- (CIMA), Tarifa, Cádiz, Spain; Sichuan University, CHINA

## Abstract

Animal-vehicle collisions have become a serious traffic safety issue. Collisions have steadily increased over the last few decades, as have their associated socio-economic costs. Here, we explore the spatial and temporal patterns of animal-vehicle collisions reported to authorities in the province of Seville, southern Spain. Most animal-vehicle collisions involved domestic animals (>95%), particularly dogs (>80%), a pattern that sharply contrasts with that found in other Spanish and European regions, where collisions are mostly caused by game species. Dog-vehicle collisions were related to the traffic intensity of the roads and they were more frequent around dawn and dusk, coinciding with the peaks of activity of dogs. This pattern was consistent throughout the week, although on weekends there were fewer collisions due to lower traffic density at those times. These findings suggest that the aggregation of dog-vehicle collisions around twilight likely resulted from a combined effect of the activity peaks of dogs and traffic density. Seasonally, collisions increased in autumn and winter, coinciding with the period of intense hunting activity in the region. Further, during autumn and winter, rush hour partly overlaps with twilight due to longer nights in comparison with summer and spring, which may contribute to the increased rate of dog-vehicle collisions in these seasons. Spatially, satellite images of nighttime lights showed that dog-vehicle collisions were clustered near urban areas. Overall, the high incidence of stray dogs involved in animal-vehicle collisions highlights a road safety issue with this type of animals in the region.

## Introduction

The number of animal-vehicle collisions (AVCs) is steadily increasing worldwide as a result of growing traffic intensities and road networks, which coincide with wildlife habitats [1–4]. Each year, AVCs cause the death of millions of animals from a wide range of taxa [5–7]. In addition to being an important source of wildlife mortality, AVCs may cause death or serious injuries to drivers, thus generating important socio-economic costs [7–9]. For example, in the United States, deer species cause 1 to 2 million collisions per year associated with 200 deaths and $8.4 billion in direct damage [10]. In 1996, Bruinderink Groot and Hazebroek estimated that more than 500,000 collisions with ungulates occurred annually in Europe, causing about 30,000 injuries and an economic cost of $1 billon [11]. However, recent studies in diverse European countries suggest that these figures are substantially higher and affect a wide range of taxa [5,6,12]. In Spain, the National Traffic Authority (DGT) reported that collisions doubled between 2006 and 2012 (on average, 12,433 AVCs per year) in comparison to those reported in 2003 (6,227 AVCs), which caused 2,911 injuries and an estimated annual cost of 105 million € [13].

A number of mitigation measures have been designed and implemented worldwide to reduce AVCs, including, among others, warning signs, wildlife crossing structures and a variety of fences, but none of these has been totally effective (reviewed in [14]). This is because patterns of AVCs may vary, for example, daily and seasonally according to the life history of the species affected (e.g., due to feeding activity or breeding). In this regards, given that implement mitigation measures along the whole road network is economically and logistically unfeasible, efforts should be focused on particularly dangerous areas. Identifying and quantifying the species affected in AVCs as well as the spatiotemporal hotspots of collisions is thus essential to develop and maximize the effectiveness of mitigation measures [14–16].

Several studies in central and northern Spain (e.g. [17–19]) have shown that ungulates cause >70% of the total collisions reported to the traffic authorities, resembling the patterns found in other countries [10,12,20–22]. Further, a study conducted at the national level in Spain yielded similar results [13], although this work was exclusively focused on collisions with wild animals i.e., reports involving domestic animals were not analyzed. However, more than 100,000 dogs and more than 30,000 cats are abandoned annually in Spain [23,24]. These figures are particularly worrying in southern Spain since Andalusia, a region (87,000 Km^2^) that completely crosses the southernmost part of Spain, is the region with the highest number of abandoned dogs in the country (16–18,000 dogs per year; [24]). Further, southern Spain, unlike other parts of the country, has been particularly impacted by loss of natural forests and the expansion of farmland habitats [25]. Thus, one may expect that game species will cause a minor fraction of AVC in the region. In contrast, given the high levels of anthropogenic modification in the landscape of southern Spain and the high estimated number of abandoned animals, it may be expected that a large proportion of AVCs is caused by domestic animals. Given the differences in life history traits among species, identifying the sources of AVCs (e.g. wild or domestic animals) and the patterns of collisions is needed to implement effective measures for road accident prevention in the region.

Here, we used collisions reported to traffic authorities in the province of Seville (Andalusia, South Spain) to characterize their spatial and temporal (daily, weekly and monthly) distribution and investigate a range of factors potentially underlying such patterns. Then, a range of suggestions are outlined for accident mitigation.

## Material and methods

### Study area

Collision data were recorded in the province of Seville (14,036 km^2^) located in Andalusia, southern Spain. According to the Nomenclature of Territorial Units for Statistics (NUTS) of the European Union, a Spanish province is classified as a NUT 3 region (population size between 150,000–800,000).

The climate in the region is Mediterranean and slightly continental, with dry and hot summers and rainy mild winters (Csa according to Köppen-Geiger climate classification; [26]). There are nearly 3,000 hours of sunlight annually with an average annual rainfall and temperature of 606 mm and 18°C, respectively (www.aemet.es/serviciosclimaticos). The province of Seville is crossed by the Río Guadalquivir from the northeast to the southwest, which establishes a fertile river valley. The valley is dominated by mountains in the North (Sierra Norte) and South (Sierra Sur) and constituted by holm, cork and gall-oak groves alternated with extensive farming constituting dehesa systems [27]. Aljarafe and Campiña areas (west and east, respectively) of the province are reserved for fields of wheat and olives groves [28]. Doñana Natural Park, a biodiversity hotspot, characterized by large areas of marshes, is situated in the south of the province. The province has a human population density around 138.3 inhabitants per square kilometer, concentrated in the extensive metropolitan area in the center and large towns in S. Norte, S. Sur, Aljarafe and Campiña. Road density (kilometer per 1,000 residents) is lower in the province of Seville (1.97) than the road network in the country as a whole (3.56).

Many of the wildlife species potentially affected by AVCs in Seville are game species. Hunting is a popular practice in Andalusia and, particularly, Seville provides many hectares of hunting land, both for small and big game hunting (see Decree 232/2007 in BOJA 158, 10 August 2007). Further, in Seville and other southern regions of the Iberian Peninsula, the use of hunting dogs is a common choice for hunters [29]. Large and small game species (mainly wild boar, red deer, red partridge and Iberian hare) are frequently hunted using several groups of hunting dogs, consisting of about 20 dogs each and different breeds, in a hunting method called “Monteria” [29]. Given that dogs are spread along the shooting area, a fraction of dogs may become lost while hunting and run over roads, thus potentially causing AVCs. Further, it is also common, although illegal, for dogs to be abandoned, e.g. if they are old or not good enough for hunting (DGT, personal communication; [23, 24]).

In Andalusia, the authorized period for hunting large and medium sized game species lasts from October to February (i.e., fox, *Vulpes vulpes*, and most wild ungulates such as wild boar, red deer, *Cervus elaphus*, and fallow deer, *Dama dama*), except for roe deer, *Capreolus capreolus*, which can be hunted from March to August. The hunting season for popular small sized species (red partridges, *Alectoris rufa*, and Iberian hares, *Lepus granatensis*) lasts from October to January. Thus, October-February maybe considered the period with the highest hunting activity in Seville by involving the authorized time for hunting most of the game species (except for roe deer) in the region. Ungulates are present in Seville in forest and grassland areas dotted with scattered trees, whereas hares and partridges are abundant in the study area and/or restocked in hunting grounds [29].

### Data base

Collision data were provided by the Provincial Directorate of Traffic of Seville (DGT). Data included 496 collisions involving identified animals (wild and domestic species) in the province of Seville in the years 2014 (n = 251) and 2015 (n = 245). The information was directly obtained from accident reports, occurred throughout the road network in Seville (highways, national and local roads), and were recorded at the location of the accident by traffic police. In addition to administrative information, reports contained the date and time of the collision, the geographical location (GPS-kilometer), the identity of the road, if there were fatalities and/or material damage and the species involved in the accident. Reports did not contain information on the road design (e.g. existence of fences, embankments). Traffic density of the surveyed roads, defined as the average number of vehicles per day, was obtained from official data at [30,31]

We are aware that the data provided by authorities maybe an underrepresentation of the number of accidents caused by animals. This is because collisions are often not reported to authorities when there are no damage or injuries (e.g., if they occurred at low speed or with small animals). Although it is not possible to determine the percentage of unreported collisions (e.g., reports do not include the size of the animals or the breed in dogs; see below), this bias is expected to be minimal in collisions caused by large vertebrates, which are the relevant species in terms of managing and implementing measures for road accident prevention. Moreover, given that reports are recorded following standardized protocols, the information provided by traffic authorities is of great utility in unraveling the composition and spatiotemporal patterns of road accidents caused by AVCs as it has been previously shown (e.g. [12,13].

### Statistical analyses

#### Temporal analyses

**Fig 1:**
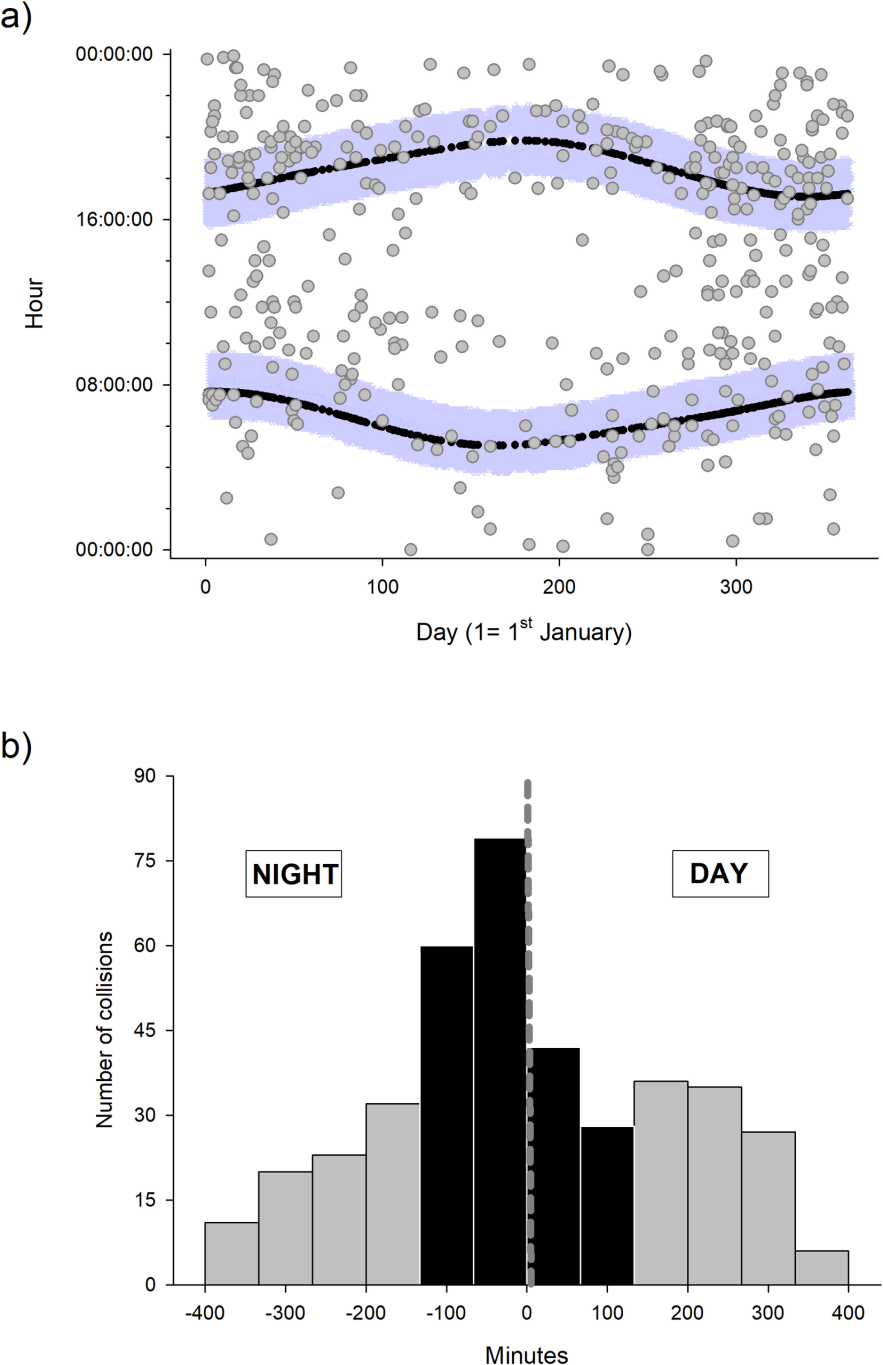
Daily and annual distribution of the dog-vehicle collisions. a) Daily and annual distribution of the dog-vehicle collisions according to solar time of the day. Black curved lines indicate time of sunrise and sunset throughout the year, whereas blue areas show the periods around twilight with the highest collision risk as shown in the bottom graph (black bars in b). b) Distribution of dog-vehicle collisions in relation to the existence of natural light. Negative values on the x-axis of the graph indicate minutes in the absence of natural light (minutes until sunrise or after sunset). Black bars show the periods during and around twilight with high concentration of dog-vehicle collisions.

**Fig 2:**
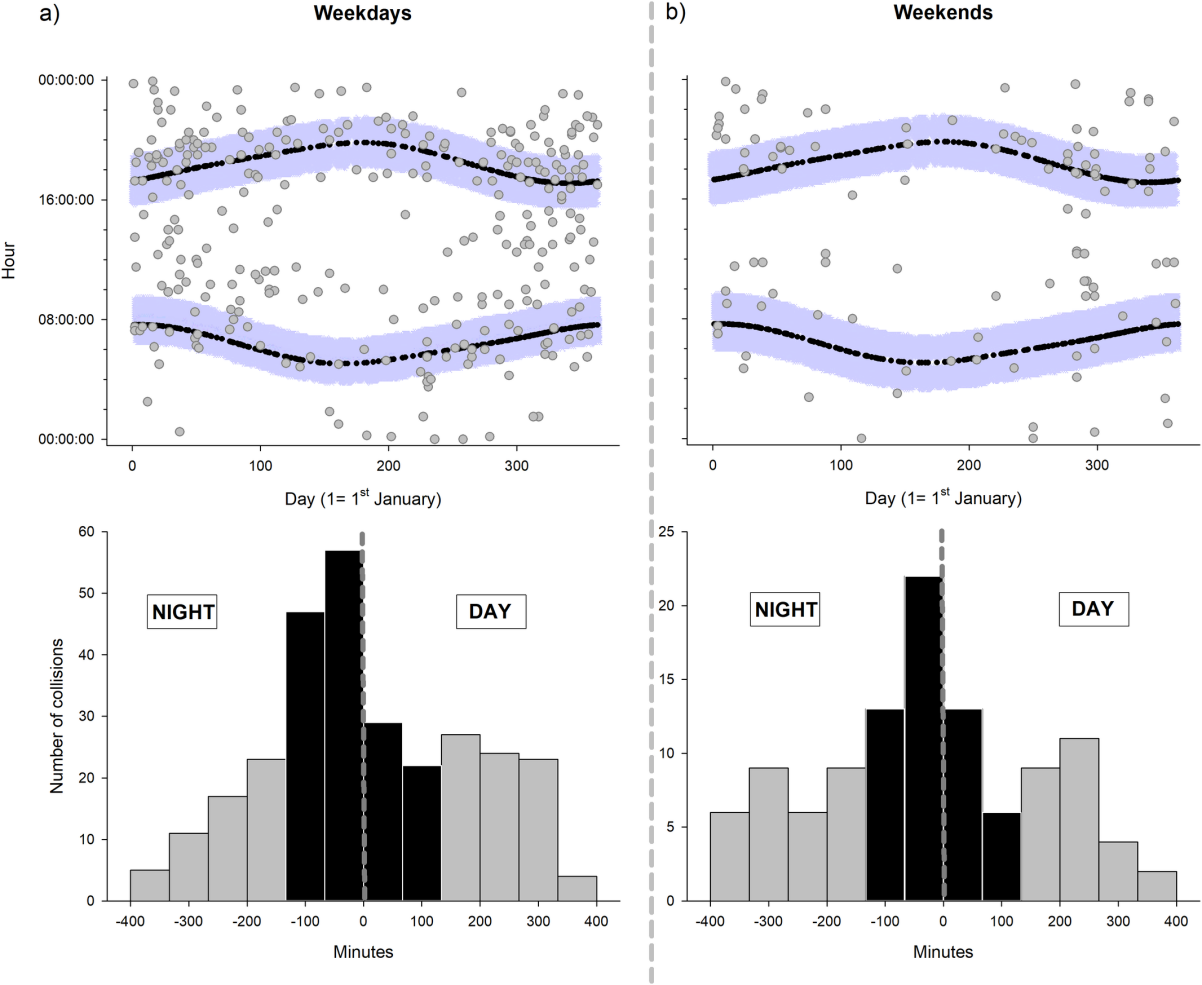
**Distribution of dog-vehicle collisions during weekdays (left side) and weekends (right side)**. Upper graphs (a, b) indicate daily and annual distribution of dog-vehicle collisions according to solar time of day. Black curved lines indicate time of sunrise and sunset throughout the year, whereas blue areas show the periods around twilight with the highest collision risk as shown in the bottom graph (black bars in c and d). Bottom graphs (c and d) show the distribution of dog-vehicle collisions in relation to the existence of natural light. Negative values on the x-axis indicate minutes in the absence of natural light (minutes until sunrise or after sunset), whereas black bars show periods during and around twilight with high concentration of dog-vehicle collisions.

We used Pearson chi-square goodness of fit to test whether the frequency of AVCs was uniformly distributed across hours of the day and months (i.e., in both cases, the null hypothesis was that these temporal categories have an equal frequency of collision). To illustrate the daily patterns of collisions in Figs 1 and 2, we obtained the times of sunrise and sunset in Seville from the Spanish Ministry of Development (http://www.fomento.gob.es/salidapuestasol/2014/Sevilla-2014.txt). We worked with solar time to avoid problems associated with changes in the official local time due to the daylight savings convention.

To test if the temporal distribution of AVCs differed between weekdays and weekends, we used Separate Kolmogorov-Smirnov (KS) two-sample tests. The KS tests were performed using the function ks.boot (10,000 simulations) in the R-package 'Matching' [32].

We used a linear model (LM) to test whether the number of AVCs, included as the dependent variable, was i) related to the monthly average length of night and ii) differed between the months with intense hunting activity (October to February) and low/no hunting activity. However, the average length of the night was also longer during the months with intense hunting activity than in the rest of the year (t = 9.042, P < 0.001), which precluded the simultaneous inclusion of both variables in a model. Consequently, only hunting season was included as a predictor in the model.

#### Spatial analyses

We fitted a generalized linear mixed (GLMM) model to test whether the probability of collision (0/1, binomial distribution) was related to the proximity to urban areas and traffic density. In this model, road identity was included as a random factor. Points without reported collisions were randomly generated (same sample size as that of collision data) across the whole road network in the region and without restrictions regarding their distance from a collision point. When measuring the distance to the nearest urban area in GIS, we realized that small urban areas, such as residential areas, were not identified in the available layers of urban areas for the region, which might bias analyses on AVCs. Thus, we used night-light levels from satellite images as a proxy of the presence of urban areas. Night-light levels were taken from a cloud-free composite of VIIRS nighttime lights produced by the Earth Observation Group, National Oceanic and Atmospheric Administration (NOAA) National Geophysical Data Center. Due to the higher spatial resolution (742 x 742 m) and no saturation, VIIRS imagery improves the DMSP satellite imagery [33]. Specifically, we used annual data for the years 2104–2015 (available at: https://ngdc.noaa.gov/eog/viirs/download_dnb_composites.html).

To further explore the relationship between traffic density and AVCs, we fitted a generalized linear model (GLM) entering the accumulated number of reported collisions in each road as the dependent variable and traffic density as predictor. We used a negative binomial rather than Poisson distribution as variance and mean of the accumulated AVCs were not equivalent [34]. To account for the influence of the road length in the number of accumulated AVCs, the length of the roads was calculated in GIS and included as an offset in the model.

Before interpreting the output of the models above, we routinely performed model diagnostics statistics to avoid misleading conclusions based on statistical artifacts. Thus, we investigated the distribution of residuals, issues about multicollinearity and the effect of influential data points. To meet statistical assumptions, traffic density and night-light levels were log_10_-transformed. After these transformations, the analyses did not show obvious deviations from the assumptions of linear models. Statistical analyses were conducted in the R 3.4.2 [35] with the packages lme4 [36], MASS [37] car [38] and DHARMa [39].

The nearest neighborhood distance (NND) method [40] was used to evaluate whether the AVCs were independently and identically distributed according to the uniform distribution over the road network, or if they followed the homogeneous binomial point process on the bounded road network. For this purpose, we tested in GIS the complete spatial randomness (CSR) hypothesis in terms of the number of roadkills in a given point set satisfying that the shortest-path distance from every roadkill to another roadkill was less than a parametric shortest-path distance [41]. For testing CSR we applied K *function method* in SANET 4.1[42].

We identified hotspots of collisions along the road network by means of a kernel density analysis using SANET V4.1 [41]. Kernel estimation is a density technique that identifies clusters by searching for dense concentrations of fatalities ([43]. Estimated densities were then classified using the Jenks method [44], based on minimization and maximization of variance respectively, within and between density classes [12].

Preliminary analyses showed that there were no interannual differences in the temporal (daily and seasonally) or spatial patterns of collisions. Thus, we show here the analyses pooling the data from the two study years.

## Results

Most of the reported traffic accidents caused by animals involved dogs (80% and 81.2% in 2014 and 2015, respectively), followed by other domestic species including horses (8% and 5.7%), sheep (3% and 1%) and cats (2% and 1.6%). Remarkably, wild boars (3% and 4.1% of total collision in 2014 and 2015, respectively) and cervids (red deer: 1% and 1.6%; no accidents involved roe deer), the species causing most of the reported accidents in other regions, had a marginal incidence in the reported collisions (S1 Table).

The high percentage of dog-vehicle collisions (DVCs, hereafter) in Seville hampered the analysis of the spatiotemporal distribution of collisions for other species or groups (i.e. domestic vs wild species). When dogs were included in the analyses, the strong influence of the species biased the results and, when excluded, the remaining sample sizes were too low to obtain reliable patterns of collisions for other species. As a consequence, further analyses were restricted to traffic accidents caused by dogs.

### Temporal analyses

**Fig 3:**
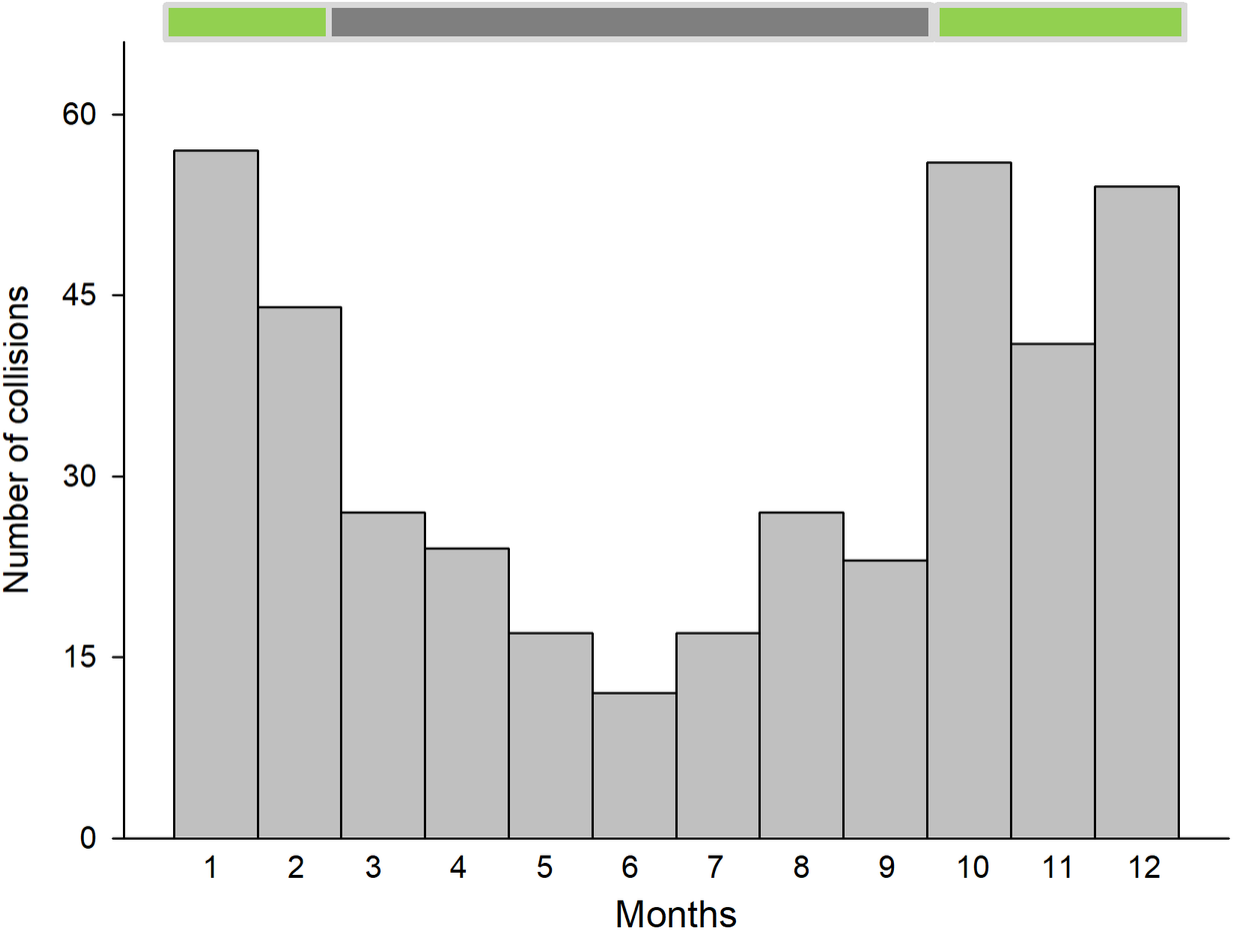
Monthly distribution of dog-vehicle collisions. The horizontal bar above the graph indicates the months with intense (green) and low/no hunting activity (grey).

Temporally, DVCs were not randomly distributed during the day (χ^2^_23_ = 128.65, P < 0.001; Fig 1). A further exploration of the data showed that DVCs were concentrated in a short period (about 75 min) before sunrise and after sunset (41% of total accidents; Fig 1B). This pattern was similar on weekends, (Kolmogorov-Smirnov test for differences in the distribution of collisions between weekdays and weekends: D = 0.10, P = 0. 34; Fig 2) although the number of collisions was lower relative to weekdays (paired t-test comparing frequency of collisions between bars from Fig 2C and 2D: t = 4.57, df = 11, P < 0.001). Across months, the frequency of DVCs was not uniformly distributed, but showed a U-shape pattern over the course of the year. DVCs increased from January to April and from October to December (χ^2^_11_ = 88.3, P < 0.001; Fig 3). The number of collisions was higher during the months with intense hunting activity (October-February; on average, 25.2 ± 0.94 collisions per month) than in the rest of the year (on average, 10.5 ± 1.59 collisions per month; LM: estimate ± 1 SE: 14.7 ± 1.74, t = 8.42, P < 0.001), although note that nights were also longer during that period of the year (see methods).

### Spatial analyses

**Fig 4:**
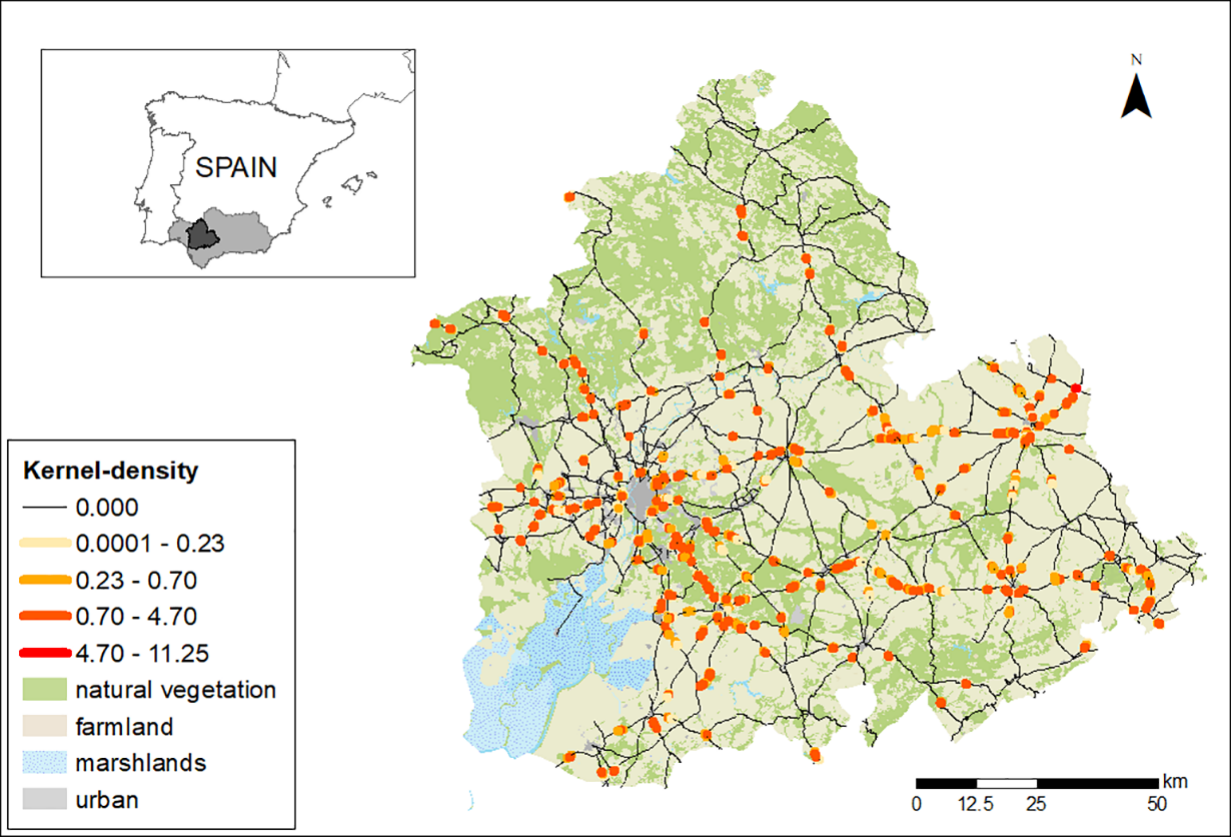
Kernel density estimation of the collision hotpots found in the road network of Seville. The land uses of the area are also shown. We used a bandwidth of 500m as the search radius for calculating the number of accidents, to estimate a density value (Natural Breaks Classification). Estimated densities classified using the Jenks method [44]. The inset map indicates the location of Seville (dark grey area) and Andalusia (light gray area) is Spain.

**Fig 5:**
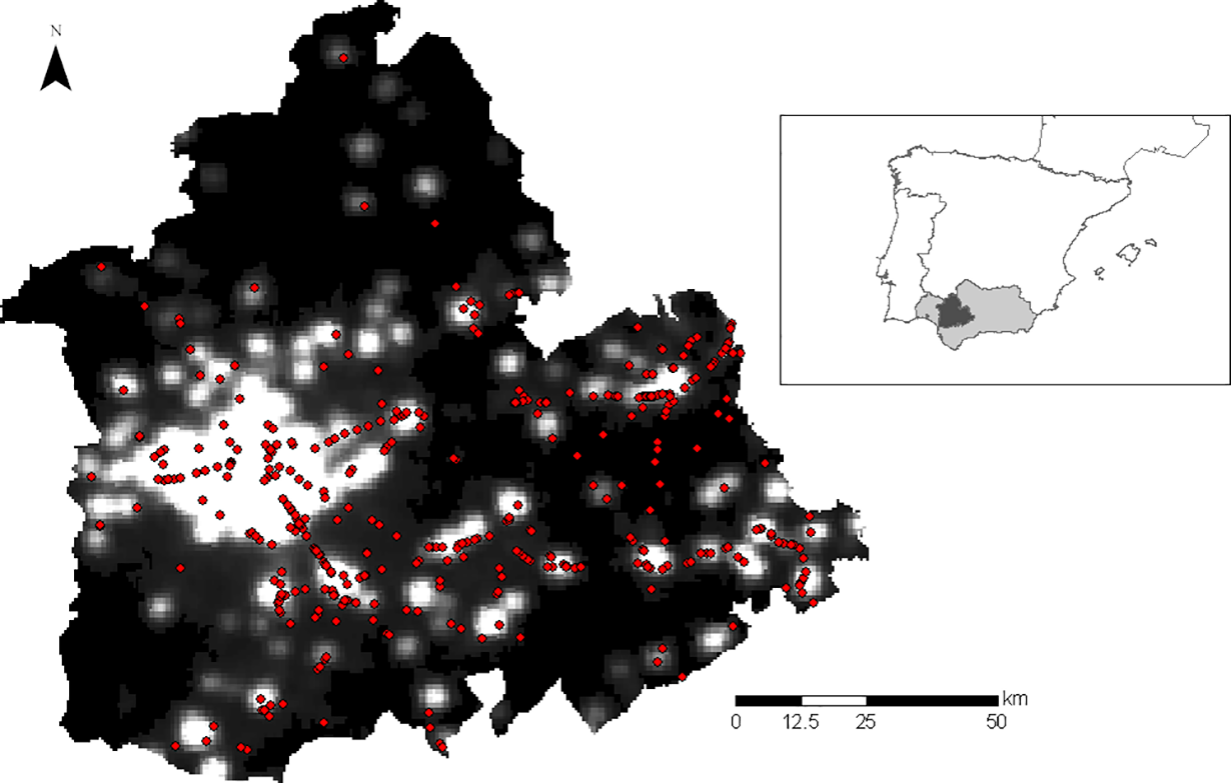
Distribution of dog-vehicle collisions in relation to night-light levels. Night-light levels were used as a proxy of the distance to urban areas (see main text for further details) in Seville. Black color indicates points without night-light, whereas white color shows points with the highest night-light levels. The inset map indicates the location of Seville (dark grey area) and Andalusia (light gray area) is Spain.

Spatially, DVCs were clustered: there were areas with a high frequency of collision as shown by kernel analyses (Fig 4). Moreover, the absence of spatial randomness (CRS) was confirmed with a 0.95 confidence level. Collision sites showed higher levels of night lights (indicating proximity to urban areas; GLMM, estimate ± 1 SE: 0.16 ± 0.05, p = 0.005) and traffic volume (estimate ± 1 SE: 0.5 ± 0.1, p< 0.001) than random points (Fig 5). In this vein, the accumulated number of collisions in a given road was also related to its traffic volume (GLM: estimate ± 1 SE: 0.33 ± 0.07, p < 0.001).

Temporal (monthly and daily) distributions of DVCs occurring inside and outside of towns and cities were similar (daily: D = 0.09, P = 0. 34; monthly: D = 0.10, P = 0. 23)

## Discussion

We found that dogs are by far the most common species reported in AVCs in the province of Seville. This finding contrasts with previous works conducted in other regions of Spain and other countries, in which ungulates are the primary group involved in reported accidents (e.g. [12,18–20]). These results are possibly a combined consequence of habitat loss and transformation occurred in South Spain during centuries [25] and the large number dogs that are abandoned annually in the region [23,24,45]. DVCs increased with traffic density and were spatially concentrated on a few roads, typically inside/near towns and cities. Temporally, DVCs were related to the activity peaks of animals (higher likelihood of collision during and around sunrise and sunset) and hunting periods.

There is substantial evidence indicating that game species, in general, and ungulates, in particular, are among the most frequent species causing mammal-vehicle collisions worldwide [7,12,20,22,46], including in Spain [13,18,19,47,48]. However, in the context of vehicle accidents caused by free-ranging animals, the incidence of domestic species as well as the factors influencing their likelihood of collision has been scarcely explored. In Spain, to our knowledge, this phenomenon has not been specifically evaluated, although the information on the total number of AVCs from previous works suggests that the rate of accidents caused by domestic animals was lower than 20–30% [18,19]. Our findings are contrary to this general trend, indicating a high incidence of dogs in AVCs reported to traffic authorities in Seville. Possibly, major transformations of the Mediterranean landscapes (including Seville), where natural woodlands have been cleared for agricultural and urban land uses [25], have led to low densities of large wild mammals (which inhabited the original natural forests) in these human-altered habitats. In fact, Seville is among the provinces of Spain with the lowest density of wild boar and roe deer and, although red deer are relatively abundant, they are mostly enclosed in fenced game estates, reducing their probability to be on roads [49,50]. Importantly, a road survey of vertebrate roadkills conducted at a larger spatiotemporal scale (10-km sections of 45 roads, regularly monitored for two years; [51]; see also Supporting Information) across Andalusia, also indicated a high incidence of DVCs. In that study, a high number of dogs (n = 93) were found killed by vehicles, constituting the most common mammal affected by roadkills after rabbits and rats, two locally common species (large mammals such as wild boars and deer were not surveyed in that study; [51]). Thus, rather than being a local phenomenon in Seville, dogs seem to be a main source of AVCs throughout southern Spain.

Numerous works have reported that the activity patterns of the species are a main factor driving the incidence of AVCs [12,19–21,52]. Accordingly, daily accidents involving dogs accumulated during and around twilight. Although the activity patterns of pets are influenced by those of human, free ranging dogs typically show crepuscular activity peaks, particularly, during summers to avoid heat [53,54]. Further, during early morning and late afternoon people may release their pets and allow them to move freely, which may increase the flush of dogs on the streets [53]. In addition to the activity pattern of animals, poor visibility conditions such as fog banks at dawn or glare from sunrise and sunset could also have contributed to shape the daily distribution of DVCs [55,56]. Further, we found strong evidence supporting a relationship between the risk of DVCs and traffic density, a factor traditionally associated with traffic accidents involving animals (birds: [57]; small mammals: [16,58]; large mammals: [22,59]). First, the probability of DVC as well as the accumulated number of DVCs per road increased with traffic density. Second, the rate of DVCs at sunrise and sunset during week days (at rush hours; [60,61]), was higher relative to that found on weekends, which are characterized by a reduced traffic density at twilight [60,61].

The high occurrence of DVCs during autumn and winter (similar results were found using the data from the study at the Andalusia level; see S1 Fig) coincides with the hunting season for most of the game species in the region, suggesting that hunting dogs (abandoned or lost while hunting) were a source of road killed animals. However, the increase in DVCs during autumn and winter could also be attributed to longer nights (poor visibility conditions) in the region during those seasons. Unfortunately, as the months with intense hunting activity and those with the longest nights overlap, it is rather difficult to disentangle the individual effect of each factor in DVCs. Further, weather conditions, with frequent rainfall and fog in autumn and winter, could also contribute to increase the rate of DVCs in these seasons by both reducing visibility and increasing the braking distance needed to avoid collisions [55].

The spatial analysis of collisions showed that DVCs predominantly occurred at sites with high levels of night-light (used as a proxy for the proximity to human settlements), suggesting that DVCs are strongly associated with towns and cities (similar results were found using the data from the study at the Andalusia level; see S1 Fig). Possibly, those accidents were caused by a combination of pets (temporally lost or permitted to move freely on the streets), true abandoned animals as well as hunting dogs that go to lighted areas searching for refuge or food once disoriented or lost. Unfortunately, there is no available information about the breed and origin of dogs involved in the reported accidents and thus these possibilities are speculative.

Overall, we have shown that DVCs were markedly clustered in time and space. The knowledge of the patterns of DVCs reported here is essential to implementing adequate mitigation measures. One possibility is the implementation of warning signals and/or informative panels, which has been proved to increase the awareness of drivers, thus reducing the probability of collision [62]. The signals should be temporary implemented during the periods (e.g. around and during twilight) and locations with the highest probability of DVCs to prevent drivers become accustomed to them [62]. However, the high incidence of dogs causing AVCs in Andalusia highlights a serious problem with the number of uncontrolled, domestic animals in the region. Although the estimates of abandoned dogs vary between studies, Seville is the province with the highest number of abandoned dogs in Andalusia (between ~16,000 [24] and ~70,000 [63] abandoned dogs per year) and Andalusia the leading region in Spain in number of abandoned dogs [24]. Thus, efforts should place priority on reducing the number of stray dogs. Campaigns should be implemented to 1) educate and raise public awareness about the social and economic consequences of accidents caused by abandoned animals e.g. multiple human trauma and costs associated with the damage to people (e.g. medical costs) and property (e.g. vehicle reparation), which are estimated in 1.4 million euros in the case of accident death; [64]. 2) Sterilize and identify dogs with chips (the latter is mandatory by the Spanish law) at a reduced cost or free of charge, especially, in rural areas. In this regard, previous studies show that only ~24% of animals entering shelters in Spain are microchipped and that microchipped pets have a lower probability of being abandoned [23,45]. 3) Capture stray animals. Additionally, our results suggest that a fraction of animals involved in DVC were used in hunting activities. Although further data is needed to quantify this phenomenon, we highlight the needed of implementing mechanisms (at the level of hunters, hunting grounds managers and rural areas) to enforce the identification of dogs, which is a current concern (DGT personal communication), as this will allow requests for possible legal liabilities in the case of DVCs. For example, in the case of hunting dogs, beside promoting programs to implant microchips, higher control efforts–scan dogs for the presence of chips and record the number of animals used per hunter—should be made by authorities before and after Monterias or during periods and days with intense hunting activity. Punitive measures maybe also applied, such as greater legal penalties for owners who abandon their dogs. Only the combined participation of all the involved parties will lead to a successful mitigation of this traffic issue.

## Supporting information

S1 Table Species involved in animal-vehicle collisions reported to authorities in the province of Seville (Spain) in the years 2014 and 2015. Collision data were provided by the Provincial Directorate of Traffic of Seville (DGT).
(PDF)

S1 Fig Distribution of dog-vehicle collisions in Andalusia. Collisions are shown in relation to (a) the month and (b) night-light levels used as a proxy of the distance to urban areas (see main text for further details). Black color indicates points without night-light whereas white color shows points with the highest night-light levels. The inset map indicates the location of Andalusia (dark gray area) is Spain. Data come from road surveys performed in 2010–2011 as a part of another study (Canal et al. 2018) on wildlife road mortality conducted at a larger spatiotemporal scale (10-km sections of 45 roads, regularly monitored during two years) in Andalusia.
(PDF)
