# Supplementary material for: Dogs are the main species involved in animal-vehicle collisions in southern Spain: Daily, seasonal and spatial analyses of collisions
Source: PLoS One. 2018 Sep 14;13(9):e0203693. doi: 10.1371/journal.pone.0203693 (PMC6157827; doi:10.1371/journal.pone.0203693)
Supplement: S1 Table — Collision data were provided by the Provincial Directorate of Traffic of Seville (DGT). (PDF) [file pone.0203693.s001.pdf]

SUPPORTING INFORMATION

S1 Table. Species involved in animal-vehicle collisions reported to authorities in the province of Seville (Spain) in the years 2014 and 2015. Collision data were provided by the Provincial Directorate of Traffic of Seville (DGT).

Species	Common name	Number of collisions
Canis familiaris	Domestic dog	399
Equus caballus	Horse	33
Ovis aries	Domestic sheep	13
Sus scrofa	Wild boar	17
Felis silvestris catus	Domestic cat	8
Cervus elaphus	Red deer	6
Vulpes Vulpes	Red fox	5
Bos taurus	Cow	4
Capra sp	Goat	4
Birds	--	2
Oryctolagus cuniculus	European rabbit	2
Lepus sp.	Hare	1
Lynx pardinus	Iberian lynx	1
Meles meles	European badger	1
