# Supplementary figures and images for: Dogs are the main species involved in animal-vehicle collisions in southern Spain: Daily, seasonal and spatial analyses of collisions
Source: PLoS One. 2018 Sep 14;13(9):e0203693. doi: 10.1371/journal.pone.0203693 (PMC6157827; doi:10.1371/journal.pone.0203693)

SUPPORTING INFORMATION

S1 Fig.

a)

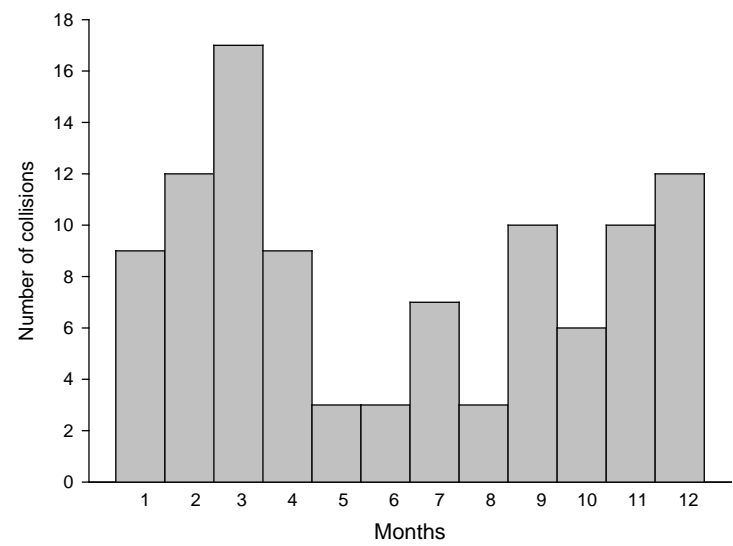

b)

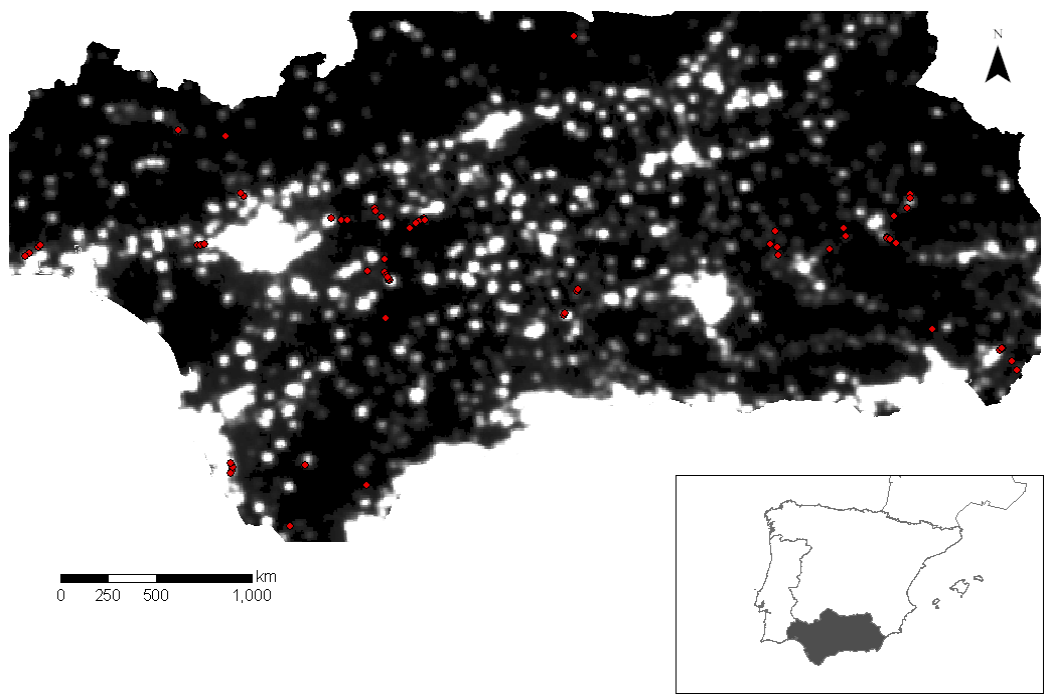

Supplement: S1 Fig — Collisions are shown in relation to (a) the month and (b) night-light levels used as a proxy of the distance to urban areas (see main text for further details). Black color indicates points without night-light whereas white color shows points with the highest night-light levels. The inset map indicates the location of Andalusia (dark gray area) is Spain. Data come from road surveys performed in 2010–2011 as a part of another study (Canal et al. 2018) on wildlife road mortality conducted at a larger spatiotemporal scale (10-km sections of 45 roads, regularly monitored during two years) in Andalusia. (PDF) [file pone.0203693.s002.pdf]
